# Object Properties Influence Visual Guidance of Motor Actions

**DOI:** 10.3390/vision3020028

**Published:** 2019-06-10

**Authors:** Sharon Scrafton, Matthew J. Stainer, Benjamin W. Tatler

**Affiliations:** 1 School of Applied Psychology, Griffith University, Gold Coast 4222, Australia; 2 School of Psychology, University of Aberdeen; Aberdeen AB24 3FX, UK

**Keywords:** eye movements, visual coordination of action, object properties, object state, visual guidance

## Abstract

The dynamic nature of the real world poses challenges for predicting where best to allocate gaze during object interactions. The same object may require different visual guidance depending on its current or upcoming state. Here, we explore how object properties (the material and shape of objects) and object state (whether it is full of liquid, or to be set down in a crowded location) influence visual supervision while setting objects down, which is an element of object interaction that has been relatively neglected in the literature. In a liquid pouring task, we asked participants to move empty glasses to a filling station; to leave them empty, half fill, or completely fill them with water; and then move them again to a tray. During the first putdown (when the glasses were all empty), visual guidance was determined only by the type of glass being set down—with more unwieldy champagne flutes being more likely to be guided than other types of glasses. However, when the glasses were then filled, glass type no longer mattered, with the material and fill level predicting whether the glasses were set down with visual supervision: full, glass material containers were more likely to be guided than empty, plastic ones. The key finding from this research is that the visual system responds flexibly to dynamic changes in object properties, likely based on predictions of risk associated with setting-down the object unsupervised by vision. The factors that govern these mechanisms can vary within the same object as it changes state.

## 1. Introduction

Accomplishing active tasks in natural environments requires human vision to be directed to appropriate areas in the environment at the time when important information is available, or is about to be available. One of the challenges that the visual system has to deal with to ensure that gaze is allocated appropriately both spatially and temporally, is that the real world is dynamic. This dynamism comes from multiple sources: from our movements through the environment, from any movements of the objects themselves in the environment and from our interactions with objects. When we interact with an object during a natural task, one common outcome is that we change some state of that object—for example, whether a box is open or closed or whether a container is empty or full. These changes of state must be accounted for when we are predicting the consequences of our actions upon objects in order to plan our actions and allocate gaze in space and time strategically: A full cup of tea requires different grip force and more careful visuomotor supervision to avoid spillage, whereas an empty cup can be maneuvered without such risks. For this flexible response to interactions with objects as we carry out our everyday activities, we are likely to draw upon a combination of currently available visual information [1] and predictions derived from internal representations about the physical world [2], for controlling action and allocating gaze appropriately [3].

In order to interact with objects in our environment, information about the locations of objects and also the functional and material properties of objects have to be extracted in order to plan actions [4]. The properties of objects affect not only our physical behavior when interacting with them but also our perceptual and visual behavior. Motor-planning research has demonstrated that the intrinsic properties of the object, such as its size, shape, surface properties and volume can have considerable effect on our grasping behavior when interacting with objects [5,6,7,8,9,10,11]. Repeated experience with objects and the consequences of our interactions with them can also be built up over prolonged periods of time, and this knowledge, based on past experience, can be used to guide behavior and eye movements. Hayhoe et al. [12] noted that participants exhibited different levels of visual guidance when extracting two different substances from receptacles, one containing jam and the other peanut butter, with the less viscous substance (jam) receiving more visual guidance during transit on the knife. Ballard and Hayhoe [13] discussed this observation and argued that the different gaze strategies, employed as a result of utilizing this type of knowledge about how different materials function in the world, demonstrates the contribution high-level information makes in gaze allocation.

The visual system is also able to adapt when properties of an object change. Hayhoe et al. [14] used a simple ball-catching task and demonstrated that with a little practice, catchers could anticipate where the ball would bounce and direct their fixation to the future bounce point approximately 53 ms before the bounce. When the ball was covertly swapped for a bouncier, faster ball, catchers took only three practices to learn the new properties of the ball and its bounce point before returning to once again making anticipatory eye movements. This result demonstrates that visual behavior is influenced by the properties and consequential behavioural functions of objects, and is adaptive to circumstances. Furthermore, the ball-catching task demonstrates the speed at which the visual system is able to take up and integrate new information about the properties of objects and plan accordingly.

During a physical interaction with an object, there are several points in time where the properties of objects may influence visual behavior. Upon the initiation of an action, vision typically leads the hand by roughly half a second [15], during which time, several types of information are available for processing that may assist the reach, pick-up and use of the object. Predictions and prior knowledge about how an object can be used—that is the affordances of objects—provide information that can influence visuomotor coordination. Indeed, while the first fixation on an object tends to be made to, or close to the object’s center of gravity [16,17], the exact location that people first fixate on will differ depending on what the individual intends to do with the object [16,18]. For example, fixations are directed to the handle of a teapot when planning to pick it up, but to the lid of the teapot when planning to open it [17].

Our interactions with man-made objects have been well-studied, primarily because man-made objects tend to be associated with rich semantic information related to their use and application [18]. Two competing accounts of how we visually attend to objects have been proposed. First, it has been suggested that when fixating on a graspable object, the viewer will look to the handle of the object [16,19,20]. Second, it has been suggested that rather than looking at the handle, the viewer looks to the action-performing part of the object [21]. Despite the qualitative and quantitative differences in viewing behavior predicted by these two accounts, the evidence as to which area is fixated upon the initiation of an interaction with an object is mixed [22].

Both of these accounts use learnt knowledge about the object and its use to strategically allocate visual attention to parts of the object. However, during an ongoing interaction with an object, the properties of that object may change. In particular, some state of the object—such as how full it is in the case of containers—may change and require different motor behavior and visual monitoring. Thus, visuomotor coordination must be flexible to the moment-to-moment changes that may occur to the objects we are interacting with, and adapt to the predicted consequences of these changes upon our behavior.

Information about the properties of objects and their consequences for visuomotor behaviour is not only important for accomplishing the beginning of actions and the subsequent execution, but also for visuomotor coordination at the end of manipulations. Commonly, after an object has been used, it is set down on a workspace or back in its original location; typically, there are two strategies of visual behavior that can accompany this type of action: either the action can be performed with or without visual guidance. The extent to which this happens appears to vary between studies, for example, in tea making only around 5% of object putdowns were visually unguided [15], whereas in sandwich making, 16% were visually unguided [23]. It is not clear what factors determine whether an object is visually monitored as we put it down or not. Land and Hayhoe [24] speculated that the difference in prevalence of guided put-downs between their tea-making and sandwich-making studies came from the fact that participants making sandwiches were seated with all objects within reach, whereas participants had to walk around the kitchen to make teas; with the latter providing much less certainty of the locations of surfaces and objects relative to the participant, and thus, having a greater need to visually guide object put-downs and less opportunity to use learning and familiarity with the immediate surroundings to reduce the need for visual guidance during putdowns. This raises the possibility that the area in which the object is being set down in may too have an influence, particularly if there are other objects present in the space (as is often the case in real life), which essentially present obstacles to avoid.

When obstacles may potentially result in collisions, people change their movement control strategy to avoid collisions [25,26,27], modifying prehensile movements adaptively depending on the presence of obstacles [28,29,30,31]. Moreover, De Haan et al. [32] demonstrated that non-spatial features of non-target objects placed in the vicinity of the reach path of a target influence behavior. Larger deviations from obstacles occurred if the obstacles had a higher consequence of collision, in this case full vs empty glasses, with the risk being higher for the full glasses. The impact of such potential consequences of collision on the visual control of actions as we set an object down is as of yet uncertain.

Understanding the temporal nature of gaze allocation in a sequential task requires us to not only investigate the fixations at the start of actions but also at the end of each individual action. The way vision is able to engage with objects during a sequential task is in part dependent on when gaze is free to move on from the previous action. In an unguided put down, vision is free to move earlier to the next object, whereas when the putdown is performed with visual guidance, the eye and hand are freed up almost simultaneously. Eye-hand latencies are an often-used measure of vision for action but studying them in isolation of visual behavior on the preceding ending action does not represent the overall picture of visual behavior in natural sequential tasks. Why some objects are guided and some not during putdowns has yet to be examined. Here, we will investigate the role of three factors that may influence visual guidance during the setting down of an object, first the inherent unchangeable properties of objects (such as the size and material); second, the properties which differ depending of the state the object is in, in this case, glasses which can either be empty or be filled with liquid to a certain level; and third, the way in which the environment influences the visual behavior during the setting down of an object, specifically whether there are potential obstacles to avoid or not. We will also consider whether the tendency to visually guide actions changes over the course of the experiment, as participants completed 108 placements of objects in order to see whether the need for visual guidance of actions changes as we become more familiar with the immediate setting in which we are acting. We hypothesize that the properties of the objects, both the inherent qualities and the changed states, and the area the object is to be set down in will produce differences in the level of visual guidance employed during the setting down of an object.

## 2. Materials and Methods

### 2.1. Participants

Five female undergraduate students from the University of Dundee took part in return for course credit. All participants had normal or corrected-to-normal vision. The study received ethical approval, and informed consent was obtained for all participants. Participants were naive to the purpose of the study.

### 2.2. Aparatus

**Figure 1:**
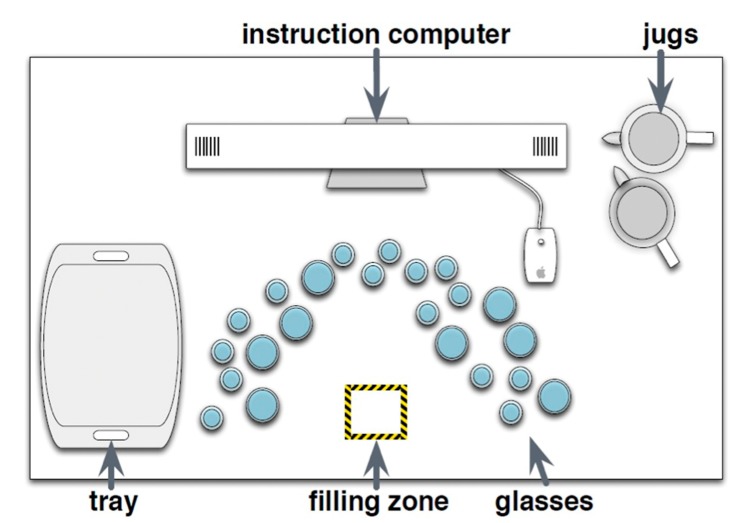
Experimental layout of workspace.

The majority of the experimental stimuli were placed on one bench-style worktop area, approximately 4 m in length. At the back of the bench was the computer displaying task instructions. The layout of the glasses, monitor, jugs and locations for filling and setting down locations can be seen below in Figure 1. In total, 44 glasses were used, (champagne flutes, high-ball, tumblers and wine glasses) all presented in both glass and plastic versions. There were six glasses of all types present in both glass and plastic, except for plastic wine glasses of which there were four. The glasses were arranged in a horseshoe shape on the workspace. Two jugs were used, one ceramic opaque and one clear glass. Ten plastic dinner trays were used as the final set down location. Instructions were presented using Matlab (v2007b) on a 20-inch iMac G5 computer, on which participants were instructed to click the mouse to receive a new set of instructions for each of the 18 trials. 

### 2.3. Eye Tracking Methodology

Eye movements were recorded using a Positive Science mobile eye tracker, sampling at 30 Hz, with a calibrated accuracy of around a degree of visual angle. The videos of the eye and scene camera were combined using Yarbus software, with eye position being estimated using feature detection (an algorithm that detects movement of an ellipse fitted to the darkest part of the video: the pupil) and the corneal reflection. Participants were calibrated using a 9-point calibration presented on a surface at the same working-height as the experiment took place.

### 2.4. Procedure

Participants were required to follow written instructions that directed them to pick up an empty glass from an array of empty glasses, place it in a designated area (the “filling zone” in Figure 1), fill it to a specified level with water from one of two jugs, and then place the glass on a tray to the side of the experimental area. The instructed fill levels were either to leave the glass empty, fill it to the top, or fill it to a half-full level. 

This sequence was repeated either four or eight times (i.e., moving, filling and placing on the tray four or eight different glasses) in each ‘trial’. For each trial, participants received instructions by clicking a computer mouse to display the full set of instructions for that trial on a computer screen positioned in front of the participant. The instructions specified for each manipulation the specific type of glass that should be selected and the level to which it should be filled with water; the instructions were left onscreen for the duration of each trial (complete tray of 4 or 8 glasses) so that participants could check back when necessary. 

After completing all four or eight instructions (glass fills and put downs) displayed on the screen for each trial, participants were asked to pick up the completed tray of glasses and move this to a separate table, where they were to empty the glasses and set the empty glasses down on the table, before returning to the task area to click for the next set of glass-filling instructions. Each participant carried out nine trials in which four glasses were manipulated and nine trials in which eight glasses were manipulated; thus 108 glasses were manipulated by each participant during the study. Of those, one-third were moved while empty, one-third were half-full and one-third were full. Within these specified experimental conditions, instructions were quasi-randomly generated for each participant using Matlab (v2007b). 

The task in total took between 40 and 60 min and two breaks were scheduled for the participant after completing blocks of six sets of instructions. During the breaks, the researcher replaced the empty glasses in the horseshoe configuration (sticker dots were arranged on the worktop marking position), the jugs were re-filled, and the participant was calibrated again before resuming the experiment. The experimental set-up and workspace is illustrated in Figure 1. 

### 2.5. Analysis

**Figure 2:**
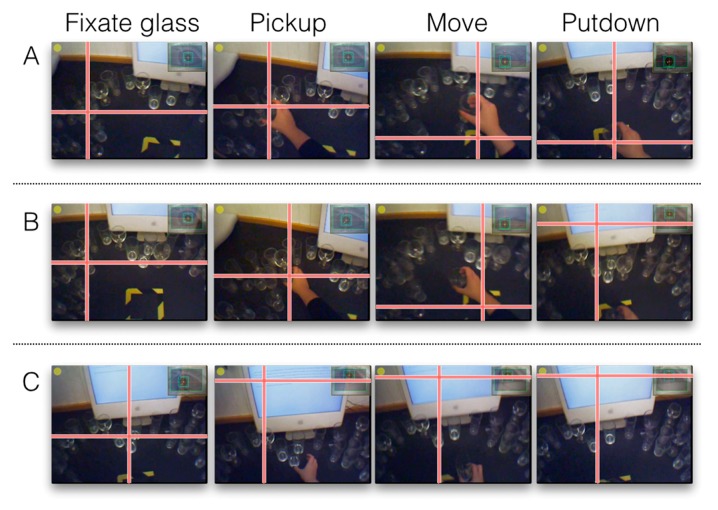
Examples of the types of object putdowns we recorded. (**A**) shows a fully-guided putdown, where the participant fixates on the glass until they pick it up, and then saccades to the setting-down location where they fixate for the entire duration of the execution of the movement and putdown of the glass. (**B**) shows a partially-guided putdown, where the visuomotor strategy is the same as (**A**), except that they saccade away from the set-down location before the put-down is complete. (**C**) shows an entirely unguided putdown where the participant fixates on the glass, then saccades back to the screen while they pick-up, and move and put-down the glass while fixating on the screen.

Gaze-fitted movies from each recording session were analysed manually on a frame-by-frame basis. For all eye movement-related measures, we recorded gaze events rather than individual fixations. Only gazes made during the putdown of objects were recorded. Object putdowns were considered either as guided, if gaze was directed either to the object or the location the object was eventually set down on, or as unguided, where no visual guidance was used for the entire setting down process. Since there are multiple ways in which one can visually guide the putting down of an object, guided putdowns were further sub-classified into fully guided if the glass or the location the glass was eventually set down on was fixated throughout the put-down, or partially guided if these locations were fixated but only for part of the action of putting down the glass. Examples of these types of behavior are shown in Figure 2.

We coded the glass used during each manipulation by type (wine glass, champagne flute, tumbler and high-ball), material, and for the second putdown state (level of liquid fill; empty, half full or full); and analyzed the type of putdown made both in the pre-manipulation state (i.e., when each glass was empty) and in post-manipulation state (empty, half-full or full) for two putdowns, one in the filling station and one on the tray.

We used a Generalised Linear Mixed-Effect Models (GLMMs) to gain better insights into the factors that govern variation in visuomotor coordination during object putdowns. For the present study, GLMMs have the advantage over ANOVA of modelling across the full dataset (i.e., every putdown). GLMMs were run using the lme4 package [33] in the R statistical analysis environment (R Core Team, 2014). The “bobyqa” optimizer was used for converging the model fits. GLMMs generate z-scores and resultant *p*-values based on the standard-normal distribution. Partial Log-odds plots were created to visualize the main effects and interactions using the remef function [34], which removes variability in the dependent variable that has been attributed to other factors in the GLMM.

## 3. Results

Participants completed all actions without error. There were no spillages of liquid, and no glasses were dropped or knocked over. Indeed, participants even correctly followed the instructions for all actions that they performed, never filling to an incorrect level or selecting the incorrect glass. This result demonstrates that participants were fully engaged with the task that they were undertaking and devoted sufficient priority so that they made no mistakes in its execution. The lack of errors may also reflect that the task asks participants to conduct action on very well-learnt visuomotor behaviour (selecting, moving, placing and filling), using objects that are very familiar to participants (glasses) from everyday experience. 

### 3.1. First Putdown

When the glasses were moved to the filling station, 37.0% of the putdowns were visually guided. A GLMM was run to predict the binary outcome of visually-guided (1) or unguided (0) putdown. Glass type (four levels), glass material (two levels) and the interaction between these two factors were included as fixed effects. In order to consider any possible learning effects across the experiment, we included trial number and glass number within each trial as fixed effects in the GLMM (to avoid overcomplicating the model we did not include any interactions between these two fixed affects and the fixed effects of glass type and material). Participant was included as a random effect, and the model with most complex random effect structure that converged is reported. Glass types (champagne flute, wine glass, high-ball and tumbler) were compared using simple coding, with champagne flute as the reference level. Material was also coded for simple contrasts, with plastic as the reference level. Using simple effects ensures that each fixed effect is evaluated at the mean of the other fixed effect rather than at the reference level of the other fixed effect as would be the case for dummy coding.

**Figure 3:**
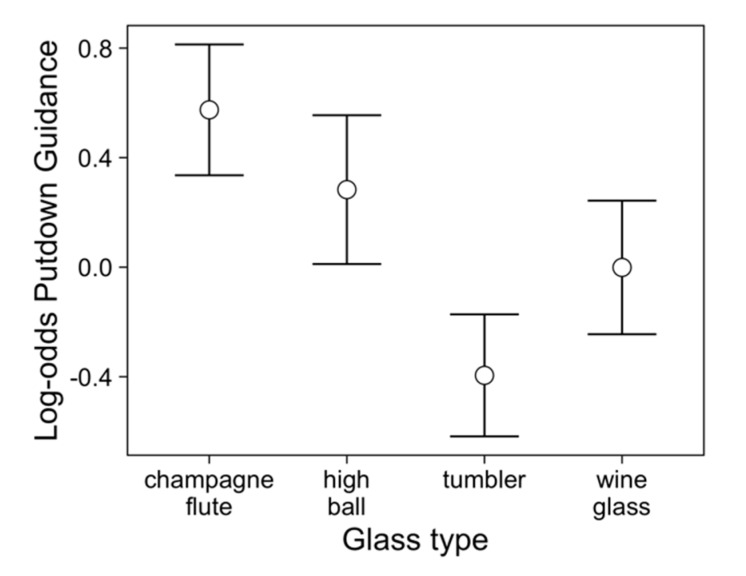
Log-odd plots of guided putdowns (for putdown 1) across participants split by glass type, with 95% confidence intervals. Higher log-odds represent a higher probability of the vessel being guided on put-down. This plot was created by removing the variability due to other factors (from the GLMM). Individual plots for each participant are shown in Appendix A Figure A1.

**Figure 4:**
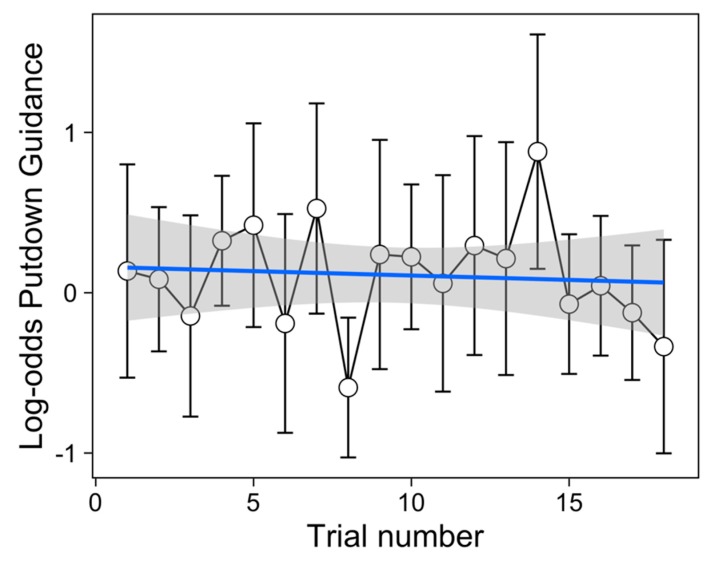
Change in GLMM calculated Log-odds of guided putdowns (for putdown 1) over trials in the experiment, with 95% CI. The linear fit is overlaid with shaded standard errors. Individual plots for each participant are shown in Appendix A Figure A2.

Table 1 shows that champagne flutes were more frequently visually guided during their setting down than both the tumblers and wine glasses (Figure 3). There was no effect of material nor was any effect (or lack thereof) of glass type modulated by the material from which it was made. There was some evidence that whether or not a putdown was visually guided decreased over trials in the experiment (Figure 4). 

### 3.2. Second Putdown

On the second glass putdown, when the glass was removed from the filling station and placed on the tray, the proportion of glasses that were guided during putdown was much larger, with 92.4% of the glasses being visually guided. A GLMM was run with glass type and material coded for simple contrasts as in the GLMM of the first putdown described earlier. Fixed effects were also included for the fullness of the glass (empty, half-full, full; coded for simple contrasts with empty as the reference level), glass number (1–8 within the trial), and trial number (1–18). As for our analysis of the first putdown, we included fixed effects describing the interaction between glass type and material, but no interaction terms involving glass number or trial number. When we attempted to include fixed effects describing the possible interaction between fill level, glass type and material, the GLMM did not converge, so no interactions between fill level and other fixed effects describing the object properties are included in the modelling that follows. The results of the most complex random effect structure that converged are reported in Table 2.

**Figure 5:**
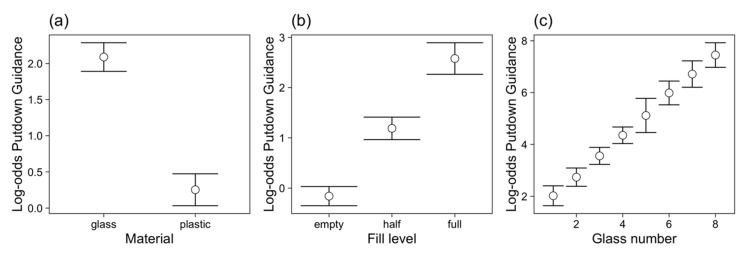
Log-odds of guided putdowns (in putdown 2) across participants to illustrate the effects of (**a**) material, (**b**) fill level and (**c**) glass (putdown) number with 95% confidence intervals. Individual plots for each participant are shown in Appendix A Figure A3.

In contrast to what was observed in the first putdown, during the second putdown material did influence whether or not the action was visually guided, with more visual guidance when placing glasses made of glass than when placing glasses made of plastic (Figure 5a). How full the glass was also influenced how frequently it was visually guided while being placed on the tray: Full glasses were more likely to be visually guided than empty ones, and half-full glasses fell between empty and full glasses in terms of how frequently they were visually guided (Figure 5b). How many glasses were already on the tray influenced whether or not the putdown was visually guided, with putdowns being increasingly more likely to be visually guided with increasing putdown number (Figure 5c). There was no change in visual guidance over the 18 trials of the experiment. 

As there was a much smaller margin for the effects to be present in, with such a high proportion of visually-guided behaviors when placing glasses on the tray, we examined whether the same factors could predict whether an object was fully visually guided (i.e., the eyes did not leave the glass or set-down location when it was being placed down) or only partially guided (i.e., the eyes left before the action was complete, or looked elsewhere during the action). This allowed us to further tease apart the demands of object and environmental properties on visual guidance strategies. We ran the same GLMM with fully guided (1) or partially guided (0) as the binary outcome variable; glass type, glass material, fill level, glass number and trial number as fixed effects, coded as in the GLMM above; and subject number as a random effect, with the model with the most complex random effect structure that converged being reported in Table 3.

**Figure 6:**
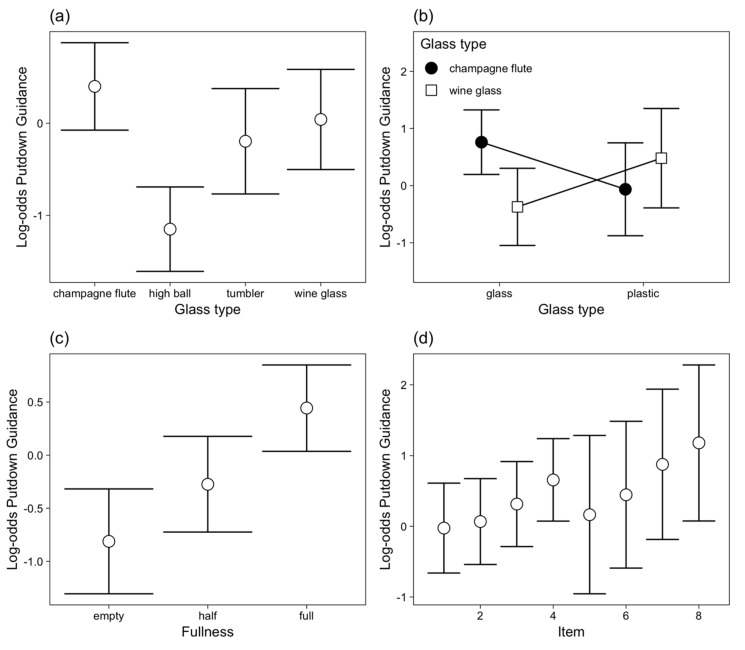
Log-odds of fully-guided (vs partially-guided) putdowns (for putdown 2) across participants to illustrate the effects of (**a**) glass type, (**b**) the interaction between glass type and material, (**c**) fill level and (**d**) glass (putdown) number with 95% CI. Individual plots for each participant are shown in Appendix A Figure A4.

Of the objects that received visual guidance during putting-down, 37.0% of the glasses were fully guided. The factors that predicted whether visually guided put downs were fully or partially guided differed slightly from those that predicted whether the action received any visual guidance (Table 3). Specifically, glass type predicted the type of visual guidance, with the champagne flute more frequently receiving visual guidance throughout the putdown than the tumblers (Figure 6a). The champagne flutes were more frequently fully visually guided than the wine glasses, but this was only the case when these were made of glass, with no difference between plastic champagne flutes and plastic wine glasses (Figure 6b). The material the glass was made from had no other influence on whether visual guidance was throughout the action or only for part of it. Full glasses were more likely to be fully guided throughout the putdown than empty glasses (Figure 6c). Whether visual guidance was full or partial also varied across glass number (1–8) on the tray, with a general increase in the frequency of fully guided putdowns as the tray became fuller (Figure 6d). Trial number did not influence whether visual guidance was full or partial.

## 4. Discussion

We found that visual monitoring of actions varied depending upon the unchanging and dynamic properties of objects and the clutter of the location in which an object was to be placed. These findings demonstrate that vision is allocated strategically to monitor the placement of objects on surfaces when there is a predicted risk associated with this putdown: when the glasses are less stable (tall glasses), more fragile (made of glass), more likely to spill their contents (full of water), or more likely to collide with another object. However, not all of these factors contributed to predicting whether or not the putdown would be guided: For two consecutive putdowns in an interaction with a single object, the factors that predicted whether or not the action would be visually guided varied considerably. This variation suggests that the properties of objects are strategically and flexibly prioritized depending upon the demands and context of each action.

We manipulated glass type and material, liquid fill level and crowding of the set down surface in order to examine the effect of object properties on visual guidance during setting down. Putdowns were more likely to be visually guided depending on the glass type, material and fill level, but this changed depending on the context. If glasses were empty, then the glass type was the most important factor that determined whether the putdown was guided or not, but when the glasses contained liquid, this along with the effect of the material from which the glass was made contributed to the decision to perform the put down with visual guidance or not. The number of glasses that were already on the put-down surface had a large effect in determining whether the glass putdown was visually guided or not: As the setting down surface became more crowded, the likelihood of visual guidance during put-down increased.

In the first putdown of each interaction with a glass, where an empty glass was placed in the empty filling station, we found a small but significant change in the extent to which this action was visually monitored over the course of the experiment, with fewer visually-guided putdowns later in the experiment. This is broadly consistent with previous work suggesting that two distinct gaze behaviours (visual guiding of a target movement or unguided target movement) are elicited as a feature of the learning process, with unguided behaviours emerging as learning of the task is acquired [35]. However, it should be noted that in our experiment the change in balance between guided and unguided putdowns over the course of the experiment was modest, and even for the first trial (the first 4 or 8 putdowns), 50% (SE = 11%) of putdowns were made without any visual guidance.

The prevalence of unguided putdowns when placing the empty glass in the filling tray, even at the start of the experiment, suggests that the ability to place objects safely on a surface does not require practice within the context of the experiment. Rather, it may be that prior knowledge of and experience with these objects may allow placements of glasses without visual supervision, drawing upon predictions based on internal models of the physical properties of objects and the relationships between objects in a scene [36]. Moreover, the effect of glass type that we found for the first putdown was present even for the first few trials of the experiment. We ran a GLMM with fixed effects of glass type and material and their interaction with the data from only the first five trials in the experiment (139 object putdowns) and found that champagne flutes were significantly more likely to be visually guided than tumblers, β = −1.16, *SE* = 0.50, *z* = −2.33, *p* = 0.020, as was found in our GLMM for all trials. The difference between champagne flutes and wine glasses found in the analysis of the full dataset was not present in the first five trials, which may reflect a lack of power of this small dataset or that it took time for this difference to emerge. The fact that the effect of glass type was found early in the experiment is consistent with the suggestion that the decision to visually supervise an object while putting it down draws upon prior knowledge of the properties of objects and the demands that they require in terms of visual guidance. However, that is not to say that we only use prior knowledge in this situation; it is more likely that we combine our prior knowledge with the present visual information in order to determine the need for visual supervision, as is demonstrated in much of the sensorimotor control literature [37,38]. The ability to combine predictions based upon prior knowledge and current visual information and update and adapt to new information is crucial for living in a dynamic world where the properties of objects can change [39].

When setting down an empty glass in an empty location (the first putdown in the sequence for our participants), the decision to use visual guidance or not seems to be based on glass type (style), with the tallest glasses, champagne flutes and high ball glasses being more likely to be guided than wine glasses or tumblers. From this result alone, it is not clear whether this might arise from the height, weight, or material of these glass types. If it was either weight or material then a difference should have been found between glass and plastic (since alongside glass being breakable, it is also heavier). However, this was not the case and there were no differences between the visual guidance of glass and plastic glasses during putdowns, when the glass was empty. From our results, it seems more likely that the deciding factor for champagne flutes and high-ball glasses to receive more visual guidance during the set down was due to height. We know from previous literature that visual behavior (fixation locations) changes with incremental increases in object height for real and computer-generated objects, both when making perceptual judgements and grasping objects [40]; thus, height influences visual behavior at the start of an action. If we consider this finding in conjunction with the results demonstrated by Cinelli, Patla, and Allard [41] showing that threat to stability (in their case when locomoting through oscillating doors) concentrates fixations on specific task-relevant features and elicits more “online” control to directly guide behavior, we can begin to appreciate that the taller glasses pose more risk in terms of their stability when being set down and, as such, require more visual guidance, hence the increase in visually-guided put downs for these glasses.

Comparing the results of the first and second putdowns of glasses in each manipulation of an object in the present study demonstrates the flexible use of object properties in determining the need for visual supervision of actions. The flexibility of behavior exhibited by the visual system has previously been well established in terms of information use and visuomotor guidance [42,43,44], and in the present study, we found that as the state of the glasses and the environment in which the action was performed changed so too did the associated visual behavior. After the initial put down, glasses had to be either left empty, half-filled, or filled, and then picked up and set down on a tray, with either four or eight glasses placed on the tray in each trial. In this setting-down scenario, the type of glass no longer mattered; instead, the important properties of the glass were the extent to which it was filled and the material it was made from. Full glasses and those made of glass were more likely to be visually guided during put down than half-full, empty or plastic glasses. The change in property of the glasses, in this case the content of liquid, affected the level of visual guidance during a put down, demonstrating that the coordination system in control of vision and action is flexible and can adapt to dynamic changes in the properties of objects. This result supports the findings of Sims, Jacobs, and Knill [43], who designed a task which imposed competing demands on the visual system using a virtual workspace environment. The task was a block-sorting task, which required vision to be used both for information acquisition and on-line guidance of a motor act. To examine visual information acquisition, blocks were rotated either 45 degrees clockwise or counter-clock wise with the aspect ratio manipulated in order to increase the difficulty of perceptual judgement. In order to examine vision during the guidance of a motor act, the size of the bins the blocks had to be placed in varied. The authors hypothesized that smaller bins would require more visual guidance to ensure accuracy. The authors found gaze to be adaptive; when the aspect ratio made the task more difficult, the block was fixated longer compared with the two easier conditions; however, less time was spent fixating on the block if the subsequent bin for placement was smaller. Sims, Jacobs, and Knill [43] argue that participants adaptively adjusted fixation allocations and durations based on the difficulty of both the task in hand and the up-coming one accordingly depending on individual varying task demands.

In comparing the two putdowns of the glasses, it should be noted that there was a higher proportion of putdowns that were unguided in the first putdown (36.97%) than in the second putdown (92.44%). This is also true when the second putdown was an empty vessel (89.74%). Why would our participants be more likely to use guidance in the second putdown if the glass was in the same state (i.e., empty)? One possibility may be that this reflects a choice of where to put the glass down. In the first putdown, the glass is always placed in the same location. In the second putdown, participants must decide *where* to best place the glass, and this decision must also reflect the location that glasses will be placed in the future and of any glasses that have already been placed on the tray. That this second putdown involves a more complex decision may account for why we observed a greater number of guided actions in the second putdown than the first. The possibility that a different context and set of factors to consider for planning the action might influence the probability that the action is visually guided is consistent with the notion that visual guidance is flexible and adaptive to the situation. 

Further support for adaptive visual behavior based on task demands is demonstrated in our finding that the level of clutter of the setting down surface contributed to the extent of visual guidance during the setting down phase. Visual guidance of the put down was more likely as the trays became fuller. As found by Sims, Jacobs, and Knill [43] once again, as an element of the task changed, the visual system responded adaptively. As the set-down area became more crowded, so too did the risk of making contact with another glass during the set down procedure, our results demonstrated that putdowns were more likely to be visually guided as the tray got fuller, indicating that we are able to adapt the level of visual guidance depending on the circumstances of the task and that our visual behavior changes as the demands of the task change.

The work of Foerster and colleagues [45] suggests that visual behaviour also changes as individuals become more experienced in a fast-paced visuomotor task. In a cup-stacking task, the allocation of fixations changed as expertise with the task was acquired across the training period. By the end of the 14-day training period, participants were found to concentrate their fixations to the top of the pyramid of stacked cups, which would subsequently be used to downstack the pile. The study also revealed that there was a fairly consistent rate of errors, for example cups falling. However, in the cup-stacking task, participants were instructed to complete the task as quickly as possible without concerns of accuracy. In the present study, not only were the participants completing a well-rehearsed common task, they also had no time pressure imposed, and indeed there were no spillages through the experiment. When performing well-rehearsed visuomotor tasks at natural temporal pace, our visual guidance seems to reflect a highly optimized coordination strategy that allows us to guide objects down to ensure that they arrive safely on a surface. 

## 5. Conclusions 

The way we visually behave when setting down an object after a manipulation has received little attention in the study of vision and eye movements, yet at this juncture in a task, the visual behavior performed carries over consequences for the next action in a sequential task, particularly in terms of at which point the eyes are free to fixate on the next object to be manipulated. Following on from initial observations in natural tasks that a significant proportion of these putdown actions are performed without visual guidance [24], we attempted to examine what it is about certain objects that may make them the subject of guidance during putdown or not. We manipulated the properties of glasses and the crowdedness of set down areas in order to try to unpack the type object properties which influence and determine the level of visual guidance used during put downs and found that the visual system is flexible in its response to changes in object properties, adapting to new circumstances and adjusting the level of guidance accordingly. Initially, the height of the object appears to determine whether the object will be guided or not, with tall glasses most likely to be visually guided during the putdown phase; however, this is only when the glass is empty. As soon as the glass is full, it is this factor along with material rather than height which demands guidance during a putdown; finally the visual system is more likely to guide putdowns as the area for setting down becomes more crowded. Considering we also see similar changes in even eye-hand latencies, with longer latencies directed towards objects with more associated risk than others, i.e., hot kettles compared with loaves of bread [46] and during the guidance of moving materials of less stability [12], it is interesting to consider whether these strategies are employed consciously or represent implicit behavioural modifications in response to the processing of an objects’ properties and associated risks. Although we cannot ascertain the extent to which these strategies are employed consciously from the present study, it has previously been established that we are often unaware of our own visual behaviour even in simple visual search tasks [47] and are inaccurate at reporting when an eye movement occurred [48]. In line with those findings, it seems likely that the mechanism behind the changes in visual strategies observed in the present study may be implicit, and individuals, if tested, would be unaware of the modifications they had made in visual guidance. The findings of the present study suggest that we utilize prior knowledge regarding the properties of objects and the way they behave in the world to inform our visual behavior and prioritize the objects with properties that demand a higher level of visual guidance, and that this is flexible and adapts to changes in properties accordingly. 

## Figures and Tables

**Table 1:** GLMM output for putdown 1, where the empty glass is placed in the filling zone. Structure of model reported here: glmer(guidance ~glass type*material + glass number + trial number + (1+ trial number||participant)).

Fixed Effect	*β*	*SE*	*z*	*p*
Glass type (High ball)	−0.39	0.30	−1.32	0.188
Glass type (Tumbler)	−0.84	0.29	−2.93	0.003 **
Glass type (Wine)	−0.58	0.29	−2.00	0.045 *
Material (Glass)	0.10	0.10	0.93	0.351
High ball:material	0.43	0.29	1.49	0.136
Tumbler:material	0.44	0.28	1.56	0.118
Wine:material	0.15	0.29	0.53	0.559
Glass number	−0.09	0.05	−1.84	0.066.
Trial number	−0.05	0.02	−1.96	0.050 *
**Random Effects Variance**				
Participants (intercept)	0.323			
Log-likelihood	−283.6			
Deviance	567.1			
AIC	591.1			
N	476			

* Significant at the 0.05 probability level. ** Significant at the 0.01 probability level.

**Table 2:** GLMM output for putdown 2, when the glass is placed on the tray. Structure of model re- ported here: glmer(guidance ~glass type*material + fullness + glass number + trial number + (1 + material + fullness + glass number||participant)).

Fixed Effect	*β*	*SE*	*z*	*p*
Glass type (High ball)	−0.31	0.67	−0.46	0.648
Glass type (Tumbler)	−0.19	0.73	−0.27	0.791
Glass type (Wine)	−0.17	0.66	−0.26	0.794
Material (Glass)	0.90	0.24	3.67	<0.001 ***
Fullness (Half-full)	1.36	0.67	2.02	0.044 *
Fullness (Full)	2.87	1.21	4.30	<0.001 ***
High ball:material	−0.77	0.64	−1.20	0.232
Tumbler:material	0.67	0.73	0.92	0.358
Wine:material	0.07	0.65	0.11	0.910
Glass number	0.79	0.18	4.30	<0.001 ***
Trial number	0.05	0.04	1.25	0.213
**Random Effects Variance**				
Participants (intercept)	<0.001			
Log-likelihood	−85.7			
Deviance	171.4			
AIC	217.4			
N	476			

* Significant at the 0.05 probability level. *** Significant at the 0.001 probability level.

**Table 3:** GLMM output for fully vs. partially visually-guided putdowns during putdown 2 (on the tray). Structure of model reported here: glmer(visual guidance type ~glass type*material + fullness + glass number + trial number + (1 + material + fullness + glass number + trial number|| participant)).

Fixed Effect	*β*	*SE*	*z*	*p*
Glass type (High ball)	−0.75	0.44	−1.69	0.090.
Glass type (Tumbler)	−1.06	0.43	−2.45	0.014 *
Glass type (Wine)	−0.23	0.41	−0.56	0.579
Material (Glass)	−0.24	0.22	−1.08	0.279
Fullness (Half-full)	0.43	0.43	1.00	0.318
Fullness (Full)	1.22	0.45	2.69	0.007 **
High ball:material	−0.55	0.44	−1.24	0.214
Tumbler:material	−0.70	0.43	−1.61	0.107
Wine:material	−1.02	0.42	−2.43	0.015 *
Glass number	0.16	0.08	2.13	0.033 *
Trial number	−0.12	0.08	−1.47	0.141
**Random Effects Variance**				
Participants (intercept)	<0.001			
Items (trays)	1.243			
Log-likelihood	−163.2			
Deviance	326.3			
AIC	386.3			
N	440			

* Significant at the 0.05 probability level. ** Significant at the 0.01 probability level.

**Figure A1:**
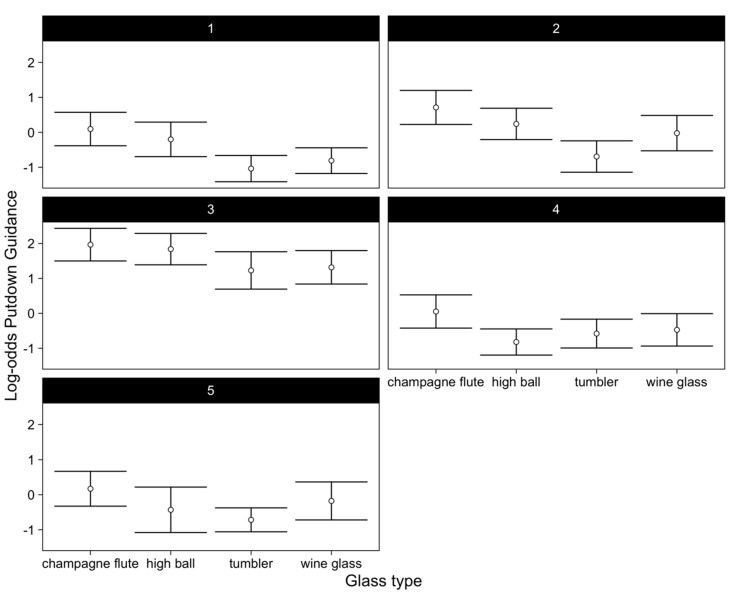
Log-odds plots of putdown 1 guidance (higher values mean the vessel was more likely to be guided during putdown), with 95% CI across glass types, with panels representing each participant.

**Figure A2:**
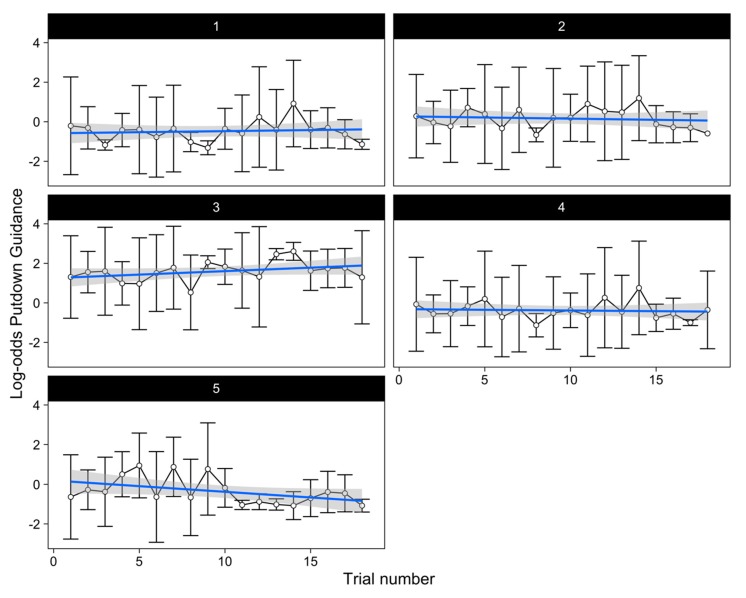
Log-odds plots of putdown 1 guidance with 95% CI across trial number, with panels representing each participant.

**Figure A3:**
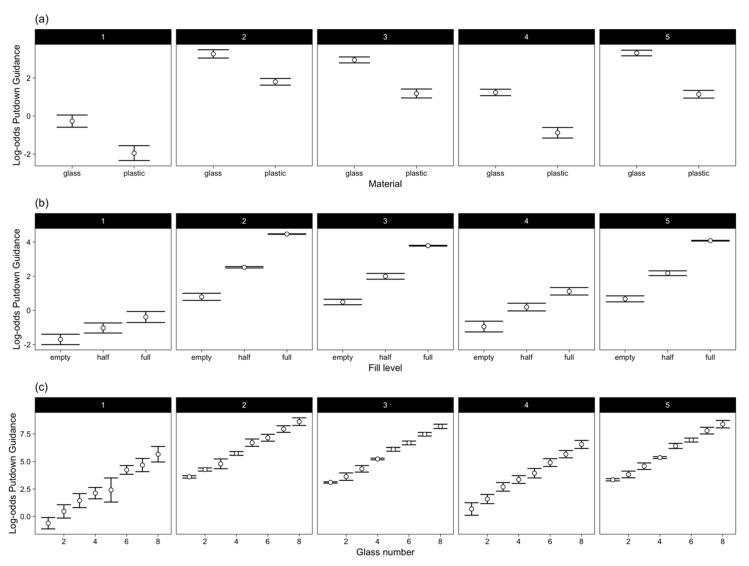
Log-odds plots of putdown 2 guidance with 95% CI across glass material (**a**), fill level (**b**) and glass number (**c**). Panels represent each participant.

**Figure A4:**
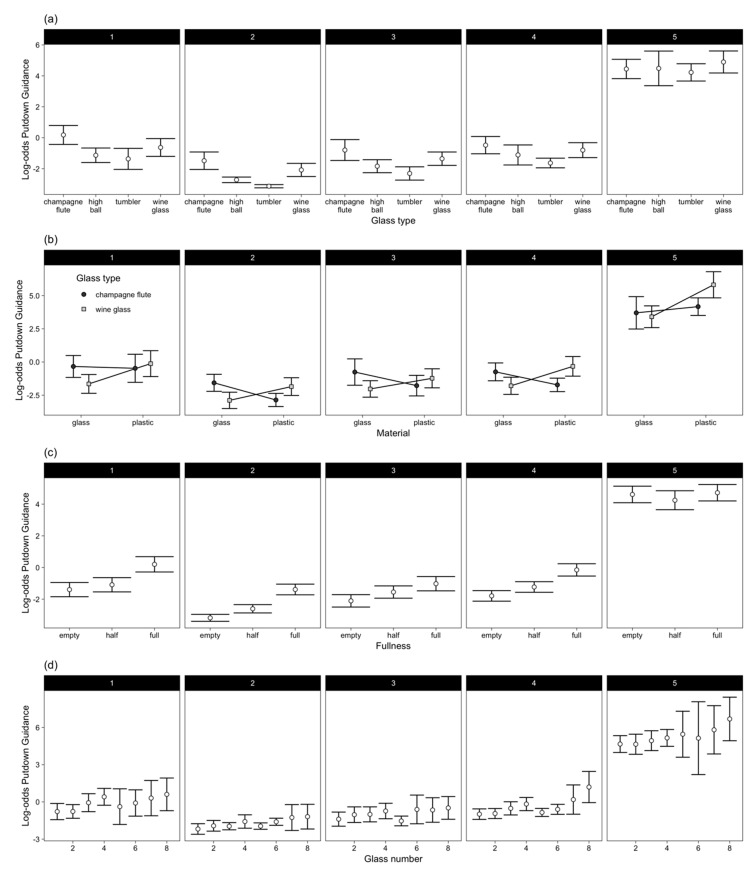
Log-odds plots of putdown 2 guidance (fully vs partially guided) with 95% CI showing the main effects of glass type (**a**), interaction between glass type and material (**b**), glass fullness (**c**) and glass number (**d**). Panels represent each participant.
